# Single-Anastomosis Sleeve Jejunal Bypass as a Treatment for Morbid Obesity: A Systematic Review and Meta-Analysis

**DOI:** 10.7759/cureus.51296

**Published:** 2023-12-29

**Authors:** Mohamad Ahmad M Alenezi, Sanad Inad H Alkhaldi, Yahya Khaled I Alrumaih, Abdullah Khalid M Alzalabani, Mohammed Ahmed M Alnujaydi, Sultan Mohammed F Alanazi, Fahad Abdullah J Alotibi, Rakan Zuwayyid A Alanazi, Malek Saad M Alanazi, Alshaymaa Akram A Alanazi

**Affiliations:** 1 Public Health Department, Maternity and Children Hospital-Arar, Ministry of Health, Arar, SAU; 2 Medical School, Faculty of Medicine, Northern Border University, Arar, SAU; 3 Medical School, Faculty of Medicine, University of Tabuk, Tabuk, SAU

**Keywords:** sleeve, single anastomosis, morbid obesity, meta-analysis, jejunum

## Abstract

Single-anastomosis sleeve jejunal (SASJ) bypass is a bariatric surgery technique with promising results. However, evidence of its efficacy and safety is still lacking. This study aimed to summarize the evidence regarding the efficacy and safety of SASJ bypass surgery in the treatment of morbid obesity. The literature was searched for English-language studies published from inception till November 26, 2023, on MEDLINE/PubMed, Cochrane Library, Web of Science, ProQuest, Scopus, SCINAPSE, and Google Scholar. The search terms included “morbid obesity,” “bariatric surgery,” and “single anastomosis sleeve jejunal bypass.” Extracted data included the body mass index (BMI) before and after surgery, percent total weight loss (%TWL), percent excess weight loss (%EWL), and improvement in preoperative comorbidities. Pooling of the data was done using random effects or fixed-effect models based on the presence of significant heterogeneity. Nine studies were included in this systematic review and meta-analysis. The change in BMI from baseline at 12 months after SASJ bypass was significant (standardized mean difference (SMD) = -3.576, 95% confidence interval (CI) = -5.423, -1.730; I² = 99.23%). At 12 months after surgery, the pooled %TWL was 42.526 (95% CI = 37.948, 47.105; I² = 97.15%), and the pooled %EWL was 75.258 (95% CI = 67.061, 83.456; I² = 99.26%). The pooled incidence of postoperative improvement in diabetes mellitus was 91% (95% CI = 79.6%, 98%, I² = 82%). The overall rate of complications was 9.9% (95% CI = 2.5%, 21.6%; I² = 92.64%). Regarding the short- and mid-term outcomes, SASJ bypass is a safe and effective procedure for weight loss in patients with morbid obesity, with an acceptable rate of complications. The procedure is also associated with a marked improvement in obesity-related comorbidities.

## Introduction and background

Obesity represents a global pandemic, affecting more than 700 million people worldwide [1]. Obesity is diagnosed when the body mass index (BMI) is 30 kg/m^2^ or above [2]. Obese individuals are at higher risk of suffering multiple chronic comorbidities, especially insulin-dependent diabetes mellitus (DM), hypertension, and obstructive sleep apnea (OSA) syndrome [3].

Several treatment lines are available for obesity. However, bariatric surgery constitutes the most successful in patients whose BMI is ≥40 or ≥35 kg/m^2^ with comorbidities [4]. Currently, the most frequently performed bariatric procedures are sleeve gastrectomy (SG), Roux-en-Y gastric bypass (RYGB), and one-anastomosis gastric bypass (OAGB) [5-7]. Nevertheless, SG has been linked with many acute postoperative complications as well as negative long-term consequences, including insufficient weight loss, weight regain, and de novo gastroesophageal reflux disease (GERD) [8-11]. In addition, RYGB requires a high level of skill and is associated with perioperative complications [12]. Furthermore, malabsorption and nutritional deficiencies have been reported following RYGB and OAGB [13,14].

Therefore, the search continues to develop new bariatric procedures or modify the existing techniques, aiming to improve patient outcomes. Single-anastomosis sleeve ileal (SASI) bypass emerged as a modification of SG with transit bipartition [15]. The SASI bypass has demonstrated several advantages, including a shorter operation time as well as permitting endoscopic evaluation of the gastrointestinal tract and biliary system [16]. Recently, single-anastomosis sleeve jejunal (SASJ) bypass was developed as an extension of the SASI bypass technique. A shorter biliopancreatic limb length is used in SASJ bypass compared to SASI bypass to improve long-term nutritional outcomes [17]. The SASJ technique is thus a promising technique that some authors claim may replace other techniques [18,19], but the available evidence needs to be evaluated to assess its safety, efficacy, and how it compares to other commonly performed techniques.

The present study was conducted to summarize the evidence regarding the efficacy and safety of SASJ bypass surgery in the treatment of morbid obesity.

## Review

Methodology

The conduction and reporting of this study followed the principles of the Cochrane Handbook for Systematic Reviews of Interventions, version 6, and the Preferred Reporting Items for Systematic Reviews and Meta-Analyses (PRISMA) guidelines [20].

Types of Included Studies

This systematic review and meta-analysis included cohort studies as well as clinical trials. The literature search was limited to studies published in the English language from inception to November 26, 2023.

Types of Excluded Studies

We excluded animal studies, case reports, conference abstracts, duplicate records, protocols, reviews, and clinical guidelines.

Participants

Eligible studies enrolled patients with morbid obesity. Morbid obesity was defined according to the criteria of the National Heart, Lung, and Blood Institutes [21] as either a BMI above 40 kg/m^2^ or a BMI above 35 kg/m^2^ in the presence of at least one medical comorbidity.

Intervention

The intervention of interest in the included studies was SASJ bypass.

Search Strategy

A literature search was carried out on the electronic databases of MEDLINE/PubMed, Cochrane Library, Web of Science, ProQuest, Scopus, SCINAPSE, and Google Scholar. The search process included all English-language articles published from inception till November 26, 2023, using the terms “morbid obesity,” “bariatric surgery,” and “single anastomosis sleeve jejunal bypass.”

Selection of Studies

We conducted the literature search, screened the titles and abstracts, retrieved the full text of apparently eligible records, and assessed the eligibility of each study for inclusion in this meta-analysis. We revised and checked the search and article selection processes.

Data Extraction

We used a standardized Excel data sheet to extract relevant data from the included studies. The extracted data included (a) the characteristics of the study (the country, study design, sample size, and follow-up); (b) patients’ characteristics (age, sex, and baseline BMI); (c) the postoperative BMI, percent total weight loss (%TWL), percent excess weight loss (%EWL), and improvement in preoperative comorbidities; and (d) SASJ bypass-related complications. We revised the data extraction process to ensure consistency.

Measured Outcomes

The primary outcomes were the change in BMI from baseline, %TWL, and %EWL at six and 12 months after SASJ bypass. The secondary outcomes include improvement in obesity-related comorbidities after SASJ bypass and the rate of complications.

Assessment of the Risk of Bias in Included Studies

For case series and non-randomized clinical trials, the risk of bias (ROB) was assessed using the methodological index for non-randomized studies (MINORS) [22]. The MINORS tool consists of 12 items: the first eight items assess single-arm studies while the other four items are used to assess comparative studies. The maximum score of MINORS is either 16 (for single-arm studies) or 24 (for comparative studies). The ROB was considered low if the score was above 12 for single-arm studies or 20 for comparative studies.

Data Synthesis

The analysis was performed using Open Meta analyst software (CEBM, Brown University, Providence, RI, USA). Significant heterogeneity of the estimates of effect was considered if the p-value from the Q statistic was below 0.10 or the I^2^ index ≥50% [23]. Pooling of the means and proportions was done according to the method of DerSimonian and Laird using a random effects model if heterogeneity was significant or the fixed-effect model in the absence of significant heterogeneity. Assessment of the publication bias was performed using Begg’s funnel plots.

Results

Results of Literature Search and Study Selection

**Figure 1:**
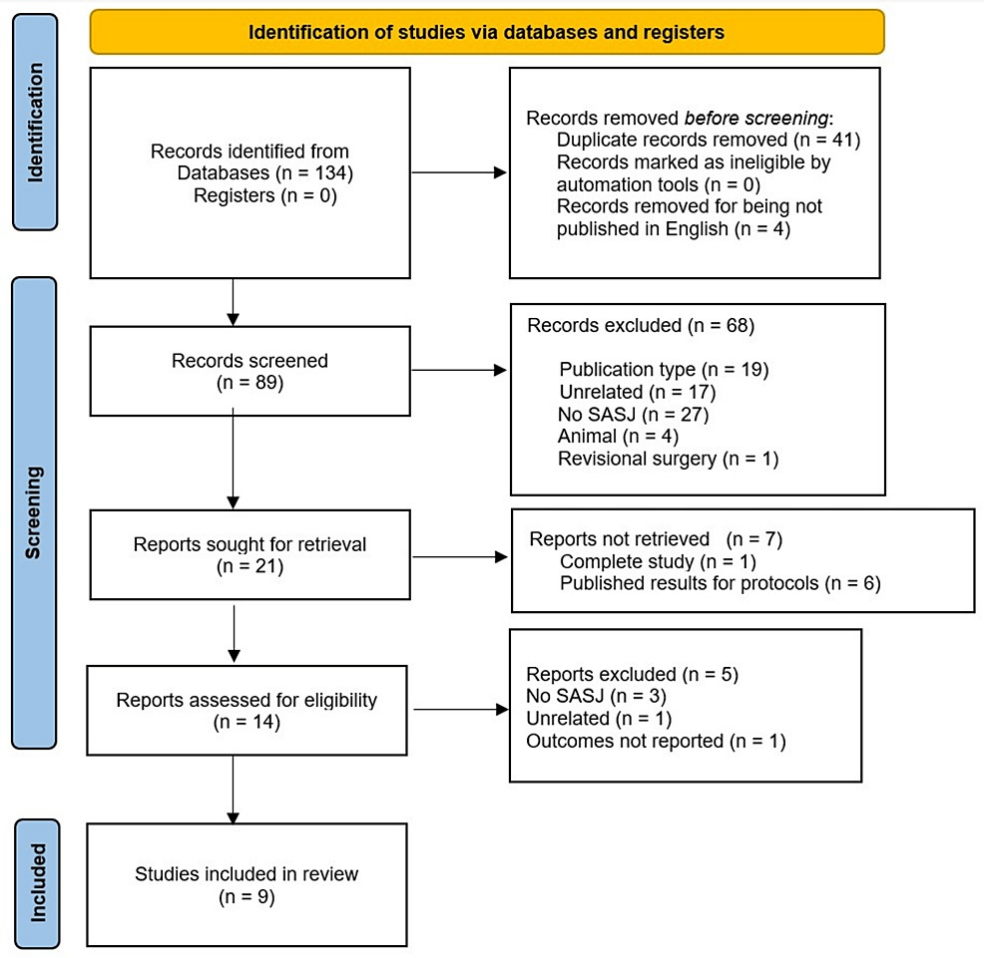
Preferred Reporting Items for Systematic Reviews and Meta-Analyses flowchart for the results of the literature search and study selection. SASJ: single-anastomosis sleeve jejunal

The literature search yielded 134 records, of which 45 were excluded (41 were duplicates and four articles were published in languages other than English). The next step was the screening of the remaining 89 records’ titles and abstracts, resulting in the exclusion of 68 records. Afterward, the full texts of 21 records were sought for retrieval, but seven records were not retrieved. The retrieved 14 full-text records were assessed for eligibility for the present meta-analysis. Only nine records were finally included [18,19,24-30] (Figure 1).

The Basic Characteristics of the Included Studies

Three studies were non-randomized clinical trials [24,28,29], two studies were randomized clinical trials [25,26], three studies were retrospective cohorts [18,27,30], and one study was a prospective cohort [19]. The studies were conducted in Iran [24,27,30] and Egypt [18,19,25,26,28,29]. Patients were mostly females, with males representing only 14-34.9% of the sample size. The follow-up duration ranged between six and 72 months (Table 1).

**Table 1:** Characteristics of the included studies (N = 9). BMI: body mass index; CT: clinical trial; NR: not recorded; OAGB: one anastomosis gastric bypass; RYGB: Roux-en-Y bypass; SD: standard deviation; SG: sleeve gastrectomy

Study	Study design	Country	Time span	Sample size (SASJ group)	Compared interventions	Follow-up (months)	Age (years), mean ± SD	Male %	Baseline BMI, mean ± SD (kg/m^2^)
Sayadishahraki et al. [24]	Non-randomized CT	Iran	January 2016 to June 2018	25	RYGB/OAGB/SG	6	NR	14%	45.26 ± 5.22
Sewefy and Saleh [19]	Prospective cohort	Egypt	April 2016 to September 2019	150	-	24	30.6 ± 7.7	28.7%	44.6 ± 4.8
Elrefai et al. [25]	Randomized CT	Egypt	December 2018 to December 2020	20	OAGB/SG	12	41.8 ± 10.4	15.0%	51.1 ± 5.8
Helmy et al. [26]	Randomized CT	Egypt	June 2019 to April 2022	100	OAGB	36	40.9 ± 12.7	30%	41.9 ± 8.7
Hosseini et al. [27]	Retrospective cohort	Iran	October 2017 to September 2021	24	SASI	12	43.4 ± 4.4	29.2%	45.19 ± 3.82
Sewefy et al. [18]	Retrospective cohort	Egypt	April 2016 to February 2021	1294	-	72	42 ± 8	29.6%	44.7 ± 4.9
Abdelzaher et al. [28]	Non-randomized CT	Egypt	November 2021 to January 2023	50	-	12	35.2 ± 11.1	28%	49.8 ± 8.3
Farrag et al. [29]	Non-randomized CT	Egypt	January 2019 to December 2019	50	OAGB	24	41.17 ± 5.3	38%	38.9 ± 6.0
Rezaei et al. [30]	Retrospective cohort	Iran	January 2016 to April 2019	43	-	18	35.6 ± 8.3	34.9%	44.9 ± 4.7

The Assessment of the Risk of Bias in the Included Studies

Overall, all studies had a high ROB. The sources of bias for cohort and non-randomized trials arose mostly from the non-clarity of whether all eligible patients were included in the study and what were the causes of exclusion [18,19,24,27,30]. In addition, none of the studies reported blinding of the patients or the assessors of outcomes [18,19,24,27-30]. Moreover, most studies did not report whether the sample size was calculated before commencing the study. The two randomized controlled trials [25,26] showed high ROB regarding the deviations from intended interventions as no information was provided on whether carers were aware of the assigned interventions. Furthermore, ROB was high regarding missing outcome data as we could not ascertain whether data were provided for all randomized patients. In addition, there was some concern in both studies regarding the selection of reported results (Table 2).

**Table 2:** Assessment of the risk of bias in the included studies (N = 9). MINORS: Q1: a clearly stated aim; Q2: inclusion of consecutive patients; Q3: prospective collection of data; Q4: endpoints appropriate to the aim of the study; Q5: unbiased assessment of the study endpoint; Q6: follow-up period appropriate to the aim of the study; Q7: loss to follow-up less than 5%; Q8: prospective calculation of the study size; Q9: an adequate control group; Q10: contemporary groups; Q11: baseline equivalence of groups; Q12: adequate statistical analyses. ROB2 domains: D1: bias arising from the randomization process; D2: bias due to deviations from intended interventions; D3: bias due to missing outcome data; D4: bias in the measurement of the outcome; D5: bias in the selection of the reported result

MINORS													
Studies	Q1	Q2	Q3	Q4	Q5	Q6	Q7	Q8	Q9	Q10	Q11	Q12	Overall ROB
Sayadishahraki et al. [24]	2	0	2	2	0	1	2	0	2	2	1	1	15 High
Sewefy and Saleh [19]	2	0	2	2	0	2	2	0	-	-	-	-	10 High
Hosseini et al. [27]	2	0	2	2	0	2	2	0	2	2	2	1	17 High
Sewefy et al. [18]	2	0	2	2	0	2	2	0	-	-	-	-	10 High
Abdelzaher et al. [28]	1	1	2	2	0	2	1	0	-	-	-	-	9 High
Farrag et al. [29]	2	2	2	2	0	2	2	0	-	-	-	-	12 High
Rezaei et al. [30]	2	0	2	2	0	2	0	0	2	2	2	1	15 High
ROB2													
Studies	D1	D2	D3	D4	D5								
Elrefai et al. [25]	Low	High	High	Low	Some concern								High
Helmy et al. [26]	High	High	High	Low	Some concern								High

Pooling of the Results of Included Studies

Three studies reported BMI at six months after surgery [24,26,30] (Table 3).

**Table 3:** Changes related to weight loss in the included studies (N = 9). All variables are summarized as mean ± standard deviation as reported by the authors. BMI: body mass index; EWL: excess weight loss; NR: not recorded; TWL: total weight loss

Study	BMI at 6 months	BMI at 12 months	%TWL at 6 months	%TWL at 12 months	%EWL at 6 months	%EWL at 12 months
Sayadishahraki et al. [24]	32.27 ± 5.63	-	NR	-	54.54 ± 14.59	-
Sewefy and Saleh [19]	NR	27 ± 1	NR	51.2 ± 14.8	NR	85 ± 11
Elrefai et al. [25]	NR	NR	39.40 ± 12.81	56.85 ± 17.04	53.47 ± 6.413	77.61 ± 9.05
Helmy et al. [26]	36.5 ± 7.2	34.2 ± 6.7	NR	NR	38.2 ± 7.4	57.4 ± 12.4
Hosseini et al. [27]	NR	30.11 ± 3.99	NR	33.78 ± 7.73	NR	76.79 ± 18.51
Sewefy et al. [18]	NR	27 ± 2	NR	39 ± 7	NR	87 ± 8
Abdelzaher et al. [28]	NR	NR	29.1 ± 4.9	44 ± 7.1	58.2 ± 11	87 ± 8.8
Farrag et al. [29]	NR	NR	NR	NR	NR	66.20 ± 8.02
Rezaei et al. [30]	32.1 ± 4.7	29.5 ± 4.5	28.7 ± 5.8	34.5 ± 6.9	54 ± 12.8	64.8 ± 15.1

**Figure 2:**
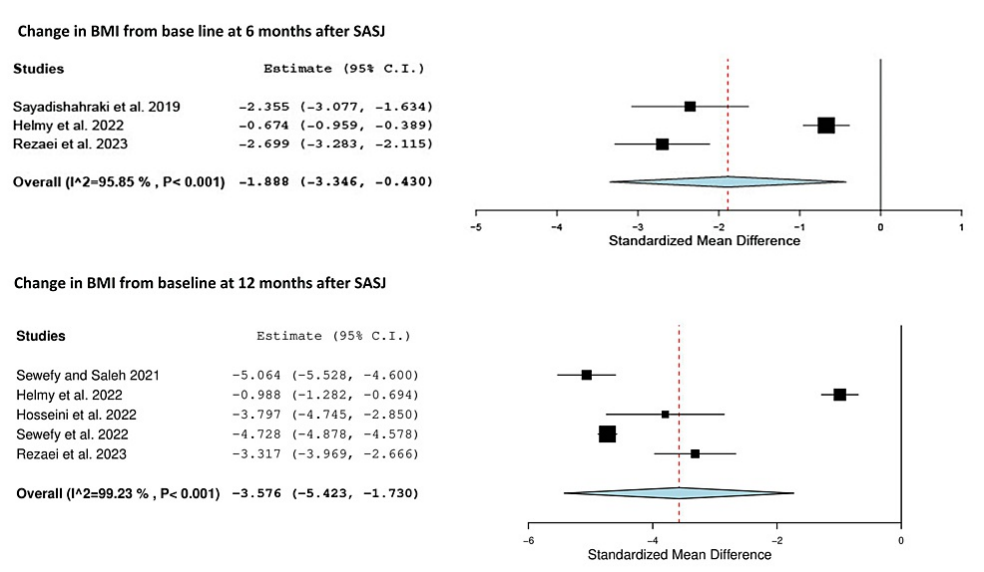
Forest plot showing the change in BMI from baseline at six and 12 months after SASJ bypass. Sayadishahraki et al. [24]; Sewefy and Saleh [19]; Helmy et al. [26]; Hosseini et al. [27]; Sewefy et al. [18]; Rezaei et al. [30]. C.I.: confidence interval; BMI: body mass index; SASJ: single-anastomosis sleeve jejunal

There was considerable heterogeneity among the studies (Q = 48.240, p < 0.001, I² = 95.85%), so the results were pooled using the random effects model. The standardized mean difference (SMD) was -1.888 (95% confidence interval (CI) = -3.346, -0.430) (Table 4, Figure 2).

**Table 4:** Heterogeneity testing and pooling of the effect size of the included studies for the assessed outcomes. BMI: body mass index; CI: confidence interval; DM: diabetes mellitus; EWL: excess weight loss; FE: fixed-effect model; GERD: gastroesophageal reflux disease; OSA: obstructive sleep apnea syndrome; RE: random effects model; SMD: standardized mean difference; TWL: total weight loss

Outcome	Studies N	Participants N	Heterogeneity testing	Model	Effect estimate [95% CI]
BMI at 6 months	3	168	Q = 48.240 (p < 0.001), I² = 95.85%	RE	SMD = -1.888 [-3.346, -0.430]
BMI at 12 months	5	1611	Q = 519.313 (p < 0.001), I² = 99.23%	RE	SMD = -3.576 [-5.423, -1.730]
%TWL at 6 months	3	113	Q = 12.972 (p = 0.002), I² = 84.58%	RE	Mean = 30.918 [27.657, 34.178]
%TWL at 12 months	6	1581	Q = 175.511 (p < 0.001), I² = 97.15%	RE	Mean = 42.526 [37.948, 47.105]
%EWL at 6 months	5	238	Q = 223.145 (p < 0.001), I² = 98.21%	RE	Mean = 51.606 [41.991, 61.222]
%EWL at 12 months	8	1731	Q = 948.263 (p < 0.001), I² = 99.26%	RE	Mean = 75.258 [67.061, 83.456]
DM improvement	8	447	Q = 38.880 (p < 0.001), I² = 82.00%	RE	Proportion = 0.910 [0.796, 0.980]
Hypertension improvement	7	503	Q = 22.611 (p < 0.001), I² = 73.46%	RE	Proportion = 0.841 [0.721, 0.931]
Hyperlipidemia improvement	5	646	Q = 16.872 (p = 0.002), I² = 76.29%	RE	Proportion = 0.931 [0.840, 0.986]
GERD improvement	4	133	Q = 0.648 (p = 0.885), I² = 0.00%	FE	Proportion = 0.881 [0.820, 0.930]
OSA improvement	5	224	Q = 5.479 (p = 0.242), I² = 27.00%	FE	Proportion = 0.993 [0.977, 1.000]
Overall complications	5	1538	Q = 54.350 (p < 0.001), I² = 92.64%	RE	Proportion = 0.099 [0.025, 0.216]

Five studies reported BMI at 12 months after SASJ [18,19,26,27,30] (Table 3). There was significant heterogeneity, so the random effects model was used to pool the results (Q = 519.313, p < 0.001, I² = 99.23%). The SMD for the difference between baseline and 12-month BMI was -3.576 (95% CI = -5.423, -1.730) (Table 4, Figure 2).

**Figure 3:**
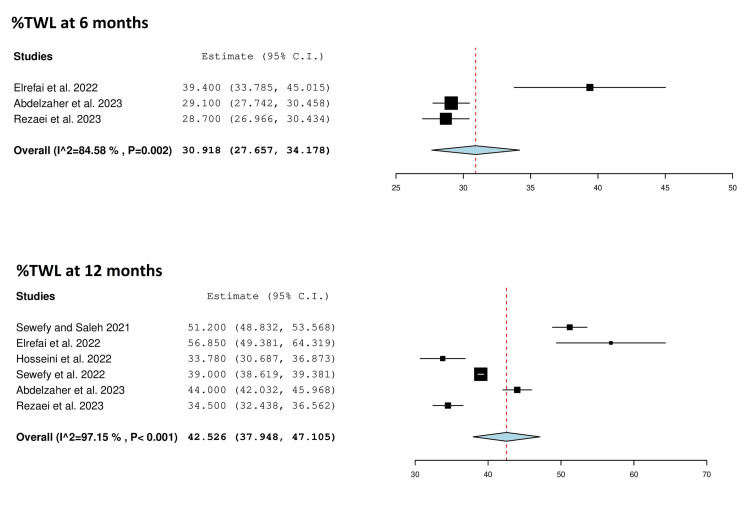
Forest plot showing the mean percentage %TWL at six and 12 months after SASJ bypass. Hosseini et al. [27]; Sewefy et al. [18]; Elrefai et al. [25]; Abdelzaher et al. [28]; Rezaei et al. [30]; Sewefy and Saleh [19]. C.I.: confidence interval; %TWL: total excess weight loss; SASJ: single-anastomosis sleeve jejunal

Three studies reported mean %TWL at six months after SASJ bypass [25,28,30] (Table 3). Significant heterogeneity existed among the studies (Q = 12.972, p = 0.002, I² = 84.58%), and the results were pooled using the random effects model. The mean %TWL was 30.918 (95% CI = 27.657, 34.178) (Table 4, Figure 3).

Six studies reported %TWL at 12 months after SASJ bypass [18,19,25,27,28,30] (Table 3). The results were pooled using the random effects model due to significant heterogeneity (Q = 175.511, p < 0.001, I² = 97.15%). The mean %TWL was 42.526 (95% CI = 37.948, 47.105) (Table 4, Figure 3).

**Figure 4:**
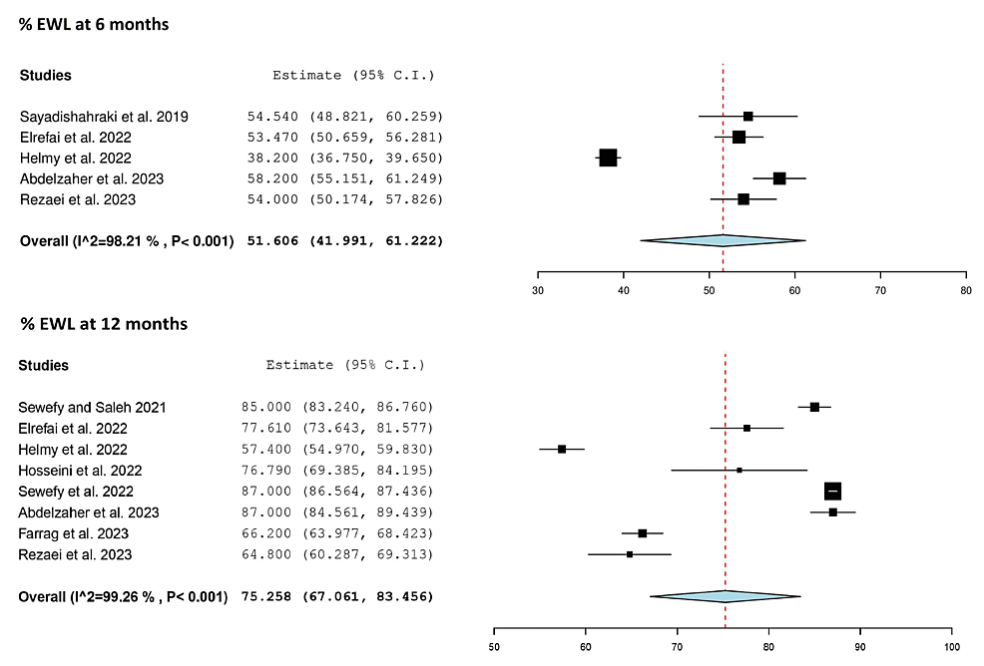
Forest plot showing the mean %EWL at six and 12 months after SASJ bypass. Sayadishahraki et al. [24]; Helmy et al. [26]; Hosseini et al. [27]; Sewefy et al. [18]; Elrefai et al. [25]; Abdelzaher et al. [28]; Rezaei et al. [30]; Sewefy and Saleh [19]; Farrag et al. [29]. C.I.: confidence interval; %EWL: percentage excess weight loss; SASJ: single-anastomosis sleeve jejunal

Five studies reported %EWL at six months after SASJ bypass [24-26,28,30] (Table 3). There was significant heterogeneity among the studies (Q = 223.145, p < 0.001, I² = 98.21%). The mean %EWL was 51.606 (95% CI = 41.991, 61.222; random effects model) (Table 4, Figure 4).

Eight studies reported %EWL at 12 months after SASJ bypass [18,19,25-30] (Table 3). Heterogeneity was significant, and the random effects model was used (Q = 948.263, p < 0.001, I² = 99.26%). The mean %EWL was 75.258 (95% CI = 67.061, 83.456) (Table 4, Figure 4).

**Figure 5:**
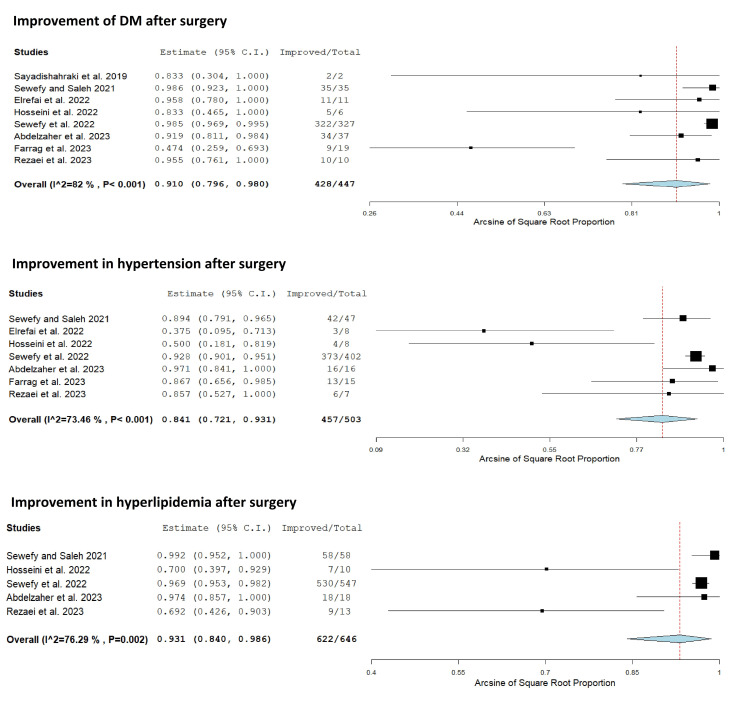
Forest plot showing the proportion of improvement of DM, hypertension, and hyperlipidemia after SASJ bypass. Sayadishahraki et al. [24]; Hosseini et al. [27]; Sewefy et al. [18]; Elrefai et al. [25]; Abdelzaher et al. [28]; Rezaei et al. [30]; Sewefy and Saleh [19]; Farrag et al. [29]. C.I.: confidence interval; DM: diabetes mellitus; SASJ: single-anastomosis sleeve jejunal

Eight studies reported an improvement in DM after SASJ bypass [18,19,24,25,27-30]. Significant heterogeneity was detected among the studies (Q = 38.880, p < 0.001, I² = 82.00%). The pooled incidence of improvement in DM was 91% (95% CI = 79.6%, 98.0%; random effects model) (Table 4, Figure 5).

Seven studies reported an improvement in hypertension after SASJ bypass [18,19,25,27-30]. Heterogeneity was significant among the studies (Q = 22.611, p < 0.001, I² = 73.46%). The pooled incidence of improvement in hypertension was 84.1% (95% CI = 72.1%, 93.1%; random effects model) (Table 4, Figure 5).

Five studies reported an improvement in hyperlipidemia after SASJ bypass [18,19,27,28,30]. Significant heterogeneity was detected among the studies (Q = 16.872, p = 0.002, I² = 76.29%). The pooled incidence of improvement in hyperlipidemia was 93.1% (95% CI = 84%, 98.6%; random effects model) (Table 4, Figure 5).

**Figure 6:**
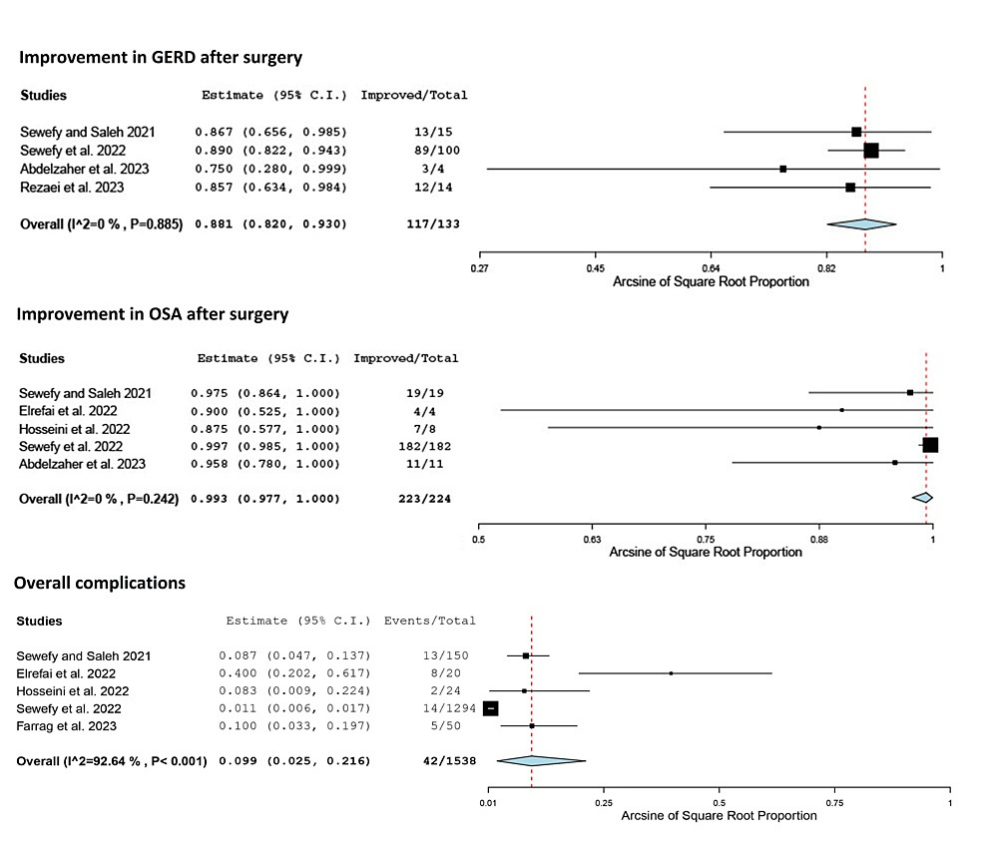
Forest plot showing the proportion of improvement of GERD and OSA syndrome, as well as overall complications after SASJ bypass. Hosseini et al. [27]; Sewefy et al. [18]; Elrefai et al. [25]; Abdelzaher et al. [28]; Rezaei et al. [30]; Sewefy and Saleh [19]; Farrag et al. [29]. C.I.: confidence interval; GERD: gastroesophageal reflux disease; OSA: obstructive sleep apnea; SASJ: single-anastomosis sleeve jejunal

Four studies reported an improvement in preoperative GERD symptoms after SASJ bypass [18,19,28,30]. No significant heterogeneity was detected among the studies (Q = 0.648, p = 0.885, I² = 0%). The pooled incidence of improvement in GERD was 88.1% (95% CI = 82%, 93%; fixed-effect model) (Table 4, Figure 6).

Five studies reported an improvement in OSA symptoms after SASJ bypass [18,19,25,27,28]. No significant heterogeneity was found among the studies (Q = 5.479, p = 0.242, I² = 27%). The pooled incidence of improvement in OSA was 99.3% (95% CI = 97.7%, 100%; fixed-effect model) (Table 4, Figure 6).

Five studies reported the rate of overall complications after SASJ bypass [18,19,25,27,29]. Significant heterogeneity existed among the studies (Q = 54.350, p < 0.001, I² = 92.64%). The pooled incidence of overall complications was 9.9% (95% CI = 2.5%, 21.6%; random effects model) (Table 4, Figure 6). The reported incidence of individual complications is detailed in Table 5.

**Table 5:** The incidence of SASJ bypass-related complications. NR: not reported; PE: pulmonary embolism; PO GERD: postoperative development of gastroesophageal reflux disease; SASJ: single-anastomosis sleeve jejunal

Study	Overall complications	Bleeding	Leak	Trocar site hernia	Excessive weight loss	Weight regain	Diarrhea	Biliary gastritis	PE	Infection	PO GERD
Sewefy and Saleh [19]	13 (8.6%)	2 (1.3%)	1 (0.7%)	NR	1 (0.7%)	1 (0.7%)	2 (1.3%)	5 (3.3%)	1 (0.7%)	NR	NR
Elrefai et al. [25]	8 (40%)	0 (0%)	0 (0%)	NR	NR	NR	NR	NR	0 (0%)	1 (5%)	0 (0%)
Helmy et al. [26]	NR	6 (6%)	2 (2%)	NR	NR	NR	NR	NR	NR	NR	3 (3%)
Hosseini et al. [27]	2 (8.2%)	1 (4.1%)	1 (4.1%)	NR	NR	NR	NR	NR	NR	NR	NR
Sewefy et al. [18]	14 (0.7%)	9 (0.5%)	1 (0.05%)	NR	NR	NR	NR	NR	1 (0.05%)	NR	NR
Abdelzaher et al. [28]	NR	2 (4%)	0 (0%)	1 (2%)	NR	NR	NR	NR	NR	2 (4%)	NR
Farrag et al. [29]	5 (10%)	2 (4%)	NR	NR	NR	NR	NR	NR	NR	NR	1 (2%)
Rezaei et al. [30]	NR	0 (0%)	0 (0%)	NR	NR	NR	NR	NR	0 (0%)	NR	NR

Comparisons Between SASJ Bypass and Other Bariatric Procedures

Five studies compared the SASJ bypass to other commonly performed bariatric procedures. Sayadishahraki et al. [24] compared SASJ bypass to RYGB, OAGB, and SG and reported the lack of significant differences among the four procedures regarding %EWL, BMI, or HbA1c at six months after surgery.

Meanwhile, Elrefai et al. [25] compared SASJ to OAGB and SG. They found that operative time was significantly longer in SASJ bypass, but no significant differences were found regarding %EWL, %TWL, complications rate, improvement of comorbidities, or quality of life. Interestingly, there was also no significant difference in diseases related to malnutrition among the assessed procedures, including iron deficiency anemia, hair loss, neuropathy, vitamin D deficiency, and hypocalcemia.

Two studies [26,29] compared SASJ and OAGB only. Both studies found that operative time was significantly longer in SASJ bypass while weight loss was significantly higher in OAGB. However, Helmy et al. [26] reported a significant difference in the resolution of DM favoring the OAGB group, while Farrag et al. [29] found no significant difference in the rate of improved comorbidities or complications between the two procedures.

Hosseini et al. [27] compared SASJ and SASI, reporting a significant decrease in mean BMI with significantly higher mean %TWL and %EWL in the SASI group. They found no significant differences between the two procedures regarding the rate of complications or laboratory measurements.

Discussion

Summary of the Main Findings

Bariatric surgery represents the most effective line of treatment for morbid obesity. Currently, several techniques have been devised. The SASJ bypass procedure is a recently developed technique that emerged as a modification of the SASI bypass procedure. The SASJ bypass is claimed to be safer than the SASI bypass as regards excessive weight loss and nutritional deficiencies. Moreover, the SASJ bypass is a simpler procedure than SASI [17].

Nine studies were retrieved for inclusion in this meta-analysis [18,19,24-30]. Most patients in the included studies were women, which accords with the published literature regarding increased demand for bariatric procedures among female patients [31].

The results of the included studies revealed a significant decrease in BMI, compared to the baseline mean values, at six and 12 months after SASJ bypass. The decrease in body weight was also evident in pooling the results of %TWL and %EWL. At six months after SASJ bypass, the pooled mean %TWL and %EWL were 30.918 (95% CI = 27.657, 34.178) and 51.606 (95% CI = 41.991, 61.222), while they reached 42.526 (95% CI = 37.948, 47.105) and 75.258 (95% CI = 67.061, 83.456), respectively, at 12 months after surgery.

Moreover, the %EWL at 12 months after SASJ in the present meta-analysis is lower than the median %EWL (90%) reported by a recent systematic review on SASI bypass [31] and is also less than that reported after RYGB (88%) [32]. However, the mean %EWL after the SASJ bypass was higher than that reported after SG (67%) [9] and was close to that reported after OAGB (72.5%) [33]. Weight loss after the SASJ bypass is attributed to both restrictive and malabsorptive mechanisms [34]. The performance of vertical gastrectomy during SASJ bypass causes early exposure of undigested food to the ileum, resulting in increased secretion of the incretin hormones, particularly glucagon-like peptide-1 which induces early satiety [35,36].

An important therapeutic effect of bariatric surgery entails the improvement of obesity-related metabolic disorders. In the current meta-analysis, the improvement rate of DM was 91%, which was slightly lower than the rate reported after the SASI bypass [31], but higher than the rates reported after SG (81.9%) [9], RYGB (70%) [37], and OAGB (83.7%) [33]. The improvement of type 2 DM after the SASJ bypass procedure could be attributed to reduced calorie intake and the rapid delivery of food to the distal bowel loops, resulting in early satiety and secretion of antihyperglycemic hormones [16].

Meanwhile, the improvement rate of hypertension was 84.1% which was higher than the rates reported after SG (66.5%) [9] and OAGB (66.94%) [33]. Moreover, hyperlipidemia improved by a mean percentage of 93.1% after the SASJ bypass compared to 64.1% after SG [9], 70% after OAGB [38], and 76.6% after the SASI bypass [31]. In addition, SASJ bypass was associated with an improvement in preoperative GERD symptoms in 88.1%, suggesting that the procedure may correct the reflexogenic effect of SG [39], probably due to the decrease in the intragastric pressure caused by adding the gastrojejunal anastomosis [40]. A similar effect was reported after the SASI bypass with an approximate rate of improvement of 92% [31].

The results of this meta-analysis revealed that SASJ bypass is a safe procedure as the rate of overall complications was 9.9%, which is close to the rate of 8.7% after SG [9] and lower than the rate of 12% after SASI bypass [31].

Overall Completeness, Applicability, and Quality of the Evidence

The present systematic review and meta-analysis summarized the current evidence on the efficacy and safety of SASJ bypass as a treatment for morbid obesity. The results of the review showed that the SASJ bypass is an effective and safe procedure for achieving excess weight loss and improving obesity-related comorbidities. However, the results of this review should be interpreted cautiously because the included studies showed several limitations. Our results were limited by the relatively small number of the included studies, most of which enrolled a small sample size. Moreover, most studies were retrospective cohorts or non-randomized clinical trials, with a high overall ROB and a relatively short period of follow-up. In addition, most studies did not provide enough details of the procedure, and the reporting of malabsorption and nutritional deficiencies was lacking in the majority of studies, thus we were not able to assess this outcome.

Another important point is the marked heterogeneity observed among the studies in most outcomes; nevertheless, we decided to present the pooled effect estimate as it was close to the median of the reported mean values and percentages. We were not able to explore the causes underlying this heterogeneity as the small number of included studies negated the performance of subgroup and/or sensitivity analyses. Our literature search yielded several protocols of randomized clinical trials that assessed and compared SASJ bypass to other bariatric procedures, but their results were not yet available. We anticipate that the inclusion of these trials after their completion and publishing in the future will allow for an update of this meta-analysis with a larger number of higher-quality studies, which could add evidence about the safety and efficacy of SASJ bypass.

## Conclusions

Regarding the short- and mid-term outcomes, SASJ bypass is a safe and effective procedure for weight loss in patients with morbid obesity, with an acceptable rate of complications. The procedure is also associated with a marked improvement in obesity-related comorbidities.

As the included studies had a high ROB, we recommend conducting large-scale randomized controlled clinical trials to compare the safety and efficacy of SASJ bypass to other commonly performed procedures of bariatric surgery. Future studies should avoid the limitations of the previous studies by calculating the sample size, ensuring effective randomization and allocation concealment of the interventions, and blinding both patients and outcome assessors to the assigned intervention. Adequate follow-up is required to assess the long-term effects of the SASJ bypass procedure.
